# Postprandial Glycemic Response to Whole Fruit versus Blended Fruit in Healthy, Young Adults

**DOI:** 10.3390/nu14214565

**Published:** 2022-10-30

**Authors:** Lisa T. Crummett, Riley J. Grosso

**Affiliations:** 1 Life Sciences, Soka University of America, Aliso Viejo, CA 92656, USA; 2 Pharmaceutical and Translational Sciences, School of Pharmacy, University of California, Los Angeles, CA 90033, USA

**Keywords:** glycemic response, postprandial, glucose, fruit, whole, blended, processed, smoothie

## Abstract

While increased intake of dietary fiber is known to reduce postprandial glycemic response, it is less understood whether the disruption of dietary fiber, in a blender, alters the postprandial glycemic response. We compared the postprandial glycemic response in 20 young, healthy college students (12 female, 8 male) after consuming whole fruit vs. blended fruit. The fruit included gala apple, with the seeds removed, and blackberries. We used a repeated measures two-way ANOVA with fruit treatment as the within-subject variable, sex as the between-subjects factor, and glucose maximum, glucose incremental area under the curve (iAUC), and 60 min glucose as dependent variables. Glucose maximum and glucose iAUC were significantly lower (*p* < 0.05) in blended fruit compared to whole fruit and 60 min glucose was marginally significantly lower (*p* = 0.057) in blended fruit compared to whole fruit. Sex was not a significant main effect and sex*treatment was not a significant interaction for any of the dependent variables. We hypothesize that a reduced glycemic response in blended apple and blackberries compared to whole apple and blackberries might be associated with the release of dietary fiber and nutritive components from ground blackberry seeds.

## 1. Introduction

Insulin resistance is the core abnormality of metabolic syndrome [1,2] a cluster of metabolic disorders that one third of adults in the United States suffer with [3], which includes: hypertension, insulin resistance, abdominal obesity, and dyslipidemia [4]. One of the main factors that promotes insulin resistance is hyperinsulinemia [5,6], which naturally follows hyperglycemia in glucose homeostasis. Thus, it is important to understand how different foods, and food processing, can promote hyperglycemia and subsequently, hyperinsulinemia, in order to follow a diet that prevents insulin resistance and ultimately, metabolic syndrome.

Increased dietary fiber intake has been associated with lower prevalence of metabolic syndrome [7]. A diet that is rich in dietary fiber reduce one’s risk of developing metabolic diseases that are associated with metabolic syndrome, including hyperlipidemia [8], cardiovascular disease [9], insulin resistance and type 2 diabetes [10,11]. Increased consumption of soluble dietary fiber has been shown to decrease postprandial glucose response and subsequently, decrease postprandial insulin response [12,13,14]. One mechanism that has been identified for how soluble dietary fiber reduces postprandial glucose and insulin response is soluble fiber increases the viscosity of ingested food [14,15]. Increased viscosity of food in the stomach reduces the rate of gastric emptying and subsequently, the absorption rate of glucose from the small intestine into circulation [14,16,17].

While it is well understood that dietary fiber mitigates postprandial spikes in glucose and insulin and protects against metabolic dysfunction, it is less understood how physical disruption of dietary fiber through mechanical processing, such as blending, might affect dietary fiber’s protective capacity. Does blended fiber have the same ability to reduce glycemic response as unprocessed dietary fiber? This is an important question to answer given that smoothies and blended fruit bowls are a popular way to consume one’s daily dose of vitamins and minerals, especially among young people, and they are commonly touted as being “healthy”. However, we know of only three studies that examined how blending fruit fiber might affect the glycemic response, one of which was published in 1977, and the results varied with the type of fruit that was used [18,19,20]. The glycemic response in some fruits (apple, mango) was not significantly different in blended form compared to whole form [18,19], whereas the glycemic response in other fruits (raspberries and passion fruit) was significantly lower in blended form compared to whole form [20]. Given that there are so few experimental studies of this nature, there is a need for additional studies to confirm previous observations and patterns, and to propose a mechanism for why the glycemic response might be altered by blending some fruits, but not others.

In this study, we compared the postprandial glycemic response in 20 young, healthy college students (12 female, 8 male), after consuming whole fruit (apple and blackberries) versus blended fruit (apple and blackberry smoothie). There are several studies that have reported sex-specific differences in postprandial glycemic response to meal treatments [21,22,23]. For this reason, we felt that it was important to include both males and females and to examine sex as a factor in our analysis. For our results to be more relevant to real-life scenarios, postprandial glycemic response was measured approximately 3.0–3.5 h after lunch, as opposed to after 12 h of fasting.

## 2. Materials and Methods

### 2.1. Participants and Study Design

Twenty healthy college students (*n* = 12 female, *n* = 8 male) from Soka University of America were recruited to participate in this study via email and in-person invitations. Participant age ranged from 19 to 21 years, with mean ± standard deviation (SD) being 20.4 ± 1 year. All participants had a non-obese body mass index (BMI) that was under 30 kg/m^2^. For the eight males, BMI ranged from 17.3 to 25.8 kg/m^2^, with a mean of 22.3 ± 2.6 kg/m^2^ (±SD); one with an underweight BMI (<18.5 kg/m^2^), six with a normal BMI (18.5 to 24.9 kg/m^2^), and one with an overweight BMI (25 to 29.9 kg/m^2^). For the 12 females, BMI ranged from 19.6 to 28.1 kg/m^2^, with a mean of 23.0 ± 3.1 kg/m^2^ (±SD); nine with a normal BMI (18.5 to 24.9 kg/m^2^), and three with an overweight BMI (25 to 29.9 kg/m^2^). Participants were prescreened such that they did not have any preexisting health conditions associated with digestion, nutrient absorption, or metabolism. A repeated measures design was used whereby all 20 participants were subjected to a whole fruit treatment and a blended fruit treatment. Experimental trials were performed from 4:00 PM to 5:10 PM. It should be noted that all participants live on campus and eat lunch at approximately the same time because there are no classes offered between 12:00 PM and 1:00 PM. Whole fruit vs. blended fruit trials were performed on different days, no more than one week apart. The whole fruit trial was performed first, followed by the blended fruit trial, to streamline food preparation. Written informed consent was obtained from each participant, and the study was approved by the Institutional Review Board at Soka University of America.

### 2.2. Fruit Treatments

One serving size of gala apple (190 g), with the central core and seeds removed and the skin included, plus one serving size of blackberries (148 g) were used in the whole fruit and blended fruit (smoothie) treatments. The blended fruit treatment was made with 100 mL of water and 100 g of ice and the total volume of the blended fruit treatment was 248 mL. To control the amount of water and ice used in the blended fruit smoothie, 200 mL of water was consumed with the whole fruit treatment. Apples and blackberries were selected for the fruit treatments because they are commonly used in fruit smoothies, and both are relatively high in dietary fiber, particularly blackberries. A single serving size of apple (190 g) contains approximately 3.23 g of insoluble fiber and 0.38 g of soluble fiber and a single serving of blackberries (148 g) contains approximately 6.66 g of insoluble fiber and 0.76 g of soluble fiber [24]. The carbohydrate percentages for gala apples are 57.2% fructose, 26.8% sucrose, and 16% glucose and the carbohydrate percentages for blackberries are approximately 50% fructose, 48% glucose, and less than 1% each of sucrose and maltose [25].

Blended apple and blended blackberry samples were incubated with invertase (Sigma I4504) for one hour at 40 °C to cleave sucrose into glucose and fructose. Then, a glucose oxidase/peroxidase assay (Protocol S1), was performed on the blended apples and blackberries to determine the total glucose present in each fruit treatment via spectrophotometer readings at 540 nm absorbance (Biotek Synergy H1 Microplate Reader) against a dextrose (Fisher D16-1) standard dilution. These enzyme assays showed that the glucose content of a gala apples was 3.3% (g/100 g) such that 190 g gala apple contained 6.27 g glucose and the glucose content of blackberries was 2.5% such that 148 g of blackberries contained 3.76 g glucose, yielding a total of 10.03 g of glucose per fruit treatment. These glucose percentages agree with reported values for apples [26] and blackberries [27].

### 2.3. Experimental Trial Procedure

On the day of an experimental trial, participants were instructed to eat lunch between 12:00 PM and 1:00 PM, three to four hours before the beginning of the experimental trial. Participants abstained from eating or drinking anything besides water between lunch and their experimental trial. After baseline glycemic measurements were taken, participants were asked to gradually consume a fruit treatment, which included 200 mL water, over the course of 10 min. After 10 min, the participant’s glycemic values were measured. Blood glucose measurements were taken every 10 min thereafter, for one hour. Blood glucose values (mg/dL) were measured in triplicate with a glucometer (Auvon Blood Glucose Monitor) and average values were used for data analysis. The accuracy of Auvon glucometers is reported to function within ±10 mg/dL of laboratory values over 95% of the time, which is beyond the International Organization for Standardization (ISO) passing standard [19]. If one of the three replicate glycemic values was 10% higher or lower than the average of the other two values, a fourth blood glucose measurement was taken to replace the outlier. During blood glucose measurements, blood was taken from the index, middle, or ring finger of either hand using a lancet fitted for the glucometer.

### 2.4. Statistical Analysis

Baseline glycemic values were subtracted from all glycemic data. The trapezoidal method was used to calculate iAUC and glycemic values that fell below baseline were converted to zero [20]. A repeated measures two-way ANOVA was performed with SPSS Statistics (version 28.0.1.0) for each of the three dependent variables: maximum blood glucose, 60 min blood glucose, and incremental area under the curve (iAUC), where fruit treatment (whole vs. blended) was the within-subjects variable and sex was the between-subjects factor. Maximum blood glucose data were square root transformed and iAUC data were log_10_ transformed to confer normality (Shapiro–Wilkinson normality test) before performing statistical tests. Statistical significance was set at *p* ≤ 0.05.

## 3. Results

### 3.1. Glycemic Response to Whole vs. Blended Fruit

**Figure 1:**
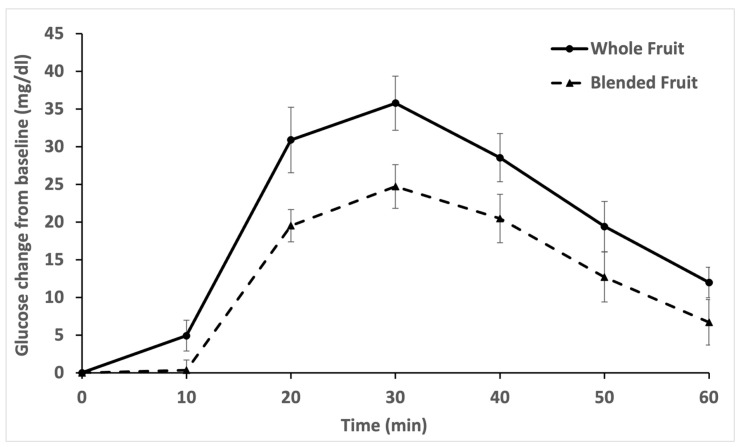
Mean incremental blood glucose values subtracted from baseline blood glucose values from 20 participants over 60 min, after consuming either whole fruit (solid line) or blended fruit (broken line). Error bars represent standard error of the mean (SEM).

**Figure 2:**
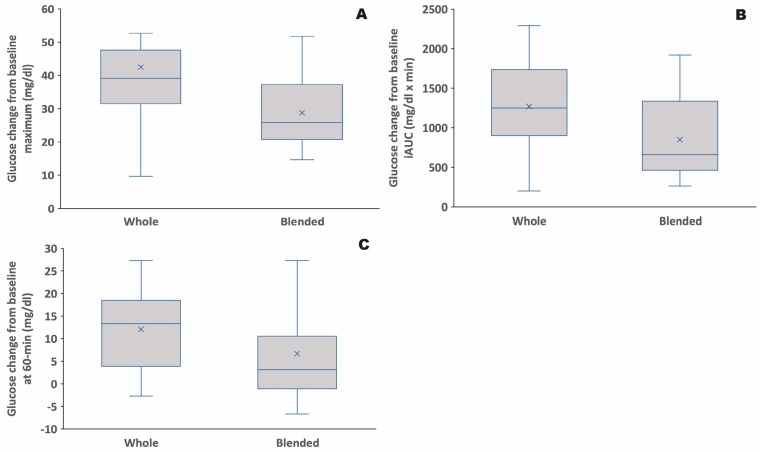
Glycemic response variables subtracted from baseline glycemic values from 20 participants after consuming whole fruit versus blended fruit, represented by (**A**) glucose maximum, (**B**) glucose incremental area under the curve (iAUC), and (**C**) incremental glucose at 60-min. Whole vs. blended fruit were significantly different (*p* ≤ 0.05) in plots (**A**,**B**) and were marginally significantly different (*p* = 0.057) in plot (**C**). Vertical bars illustrate maximum and minimum values, the top and bottom of the box represent third quartile and first quartile values, respectively, the line inside the box represents the median, and the “×” represents the mean.

Processing apple and blackberries in a blender significantly reduced the glycemic response (Figure 1; Table 1). All glycemic values are reported as differences from the baseline glycemic value. Baseline glycemic values were in the normal range for each fruit treatment (mean ± standard error of the mean (SEM): 102.4 ± 1.8 mg/dL for whole fruit vs. 105.7 ± 1.8 mg/dL for blended fruit; Figure S1). Glucose maximum was significantly lower for blended fruit compared to whole fruit (Table 1; Figure 2A; *p* = 0.004; mean ± SEM: 42.5 ± 4.6 mg/dL for whole fruit vs. 28.8 ± 2.4 mg/dL for blended fruit). Glucose incremental area under the curve (iAUC) was significantly lower for blended fruit compared to whole fruit (Table 1; Figure 2B; *p* = 0.005; mean ± SEM: 1269 ± 124 mg/dL × min for whole fruit vs. 850 ± 109 mg/dL × min for blended fruit). Glucose at 60 min was marginally significantly lower for blended fruit compared to whole fruit (Table 1; Figure 2C; *p* = 0.057; mean ± SEM: 12.1 ± 2.0 mg/dL for whole fruit vs. 6.7 ± 3.1 mg/dL for blended fruit).

### 3.2. Sex as a Factor in Glycemic Response to Whole vs. Blended Fruit

**Figure 3:**
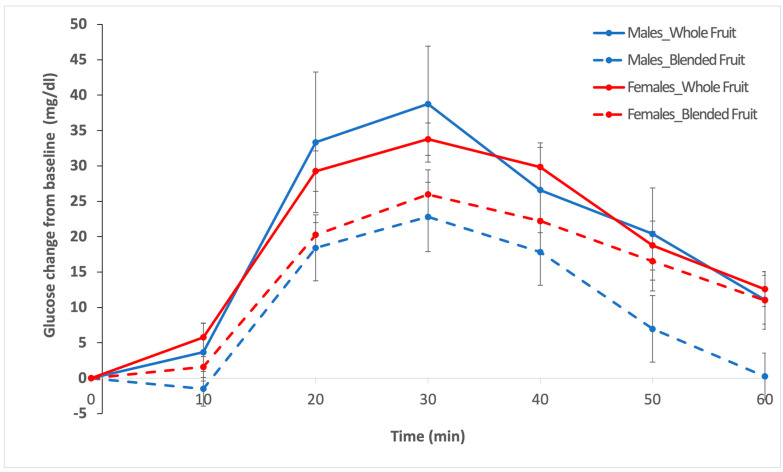
Mean incremental blood glucose values subtracted from baseline blood glucose values in males (*n* = 8; blue lines) and females (*n* = 12; red lines) over 60 min, after consuming either whole fruit (solid lines) or blended fruit (broken lines). Error bars represent SEM.

Sex did not have a statistically significant effect on glycemic response to whole fruit vs. blended fruit, based on the three dependent variables that were measured (Table 1; *p* > 0.05). Sex, as a main effect, was not significant, nor was the interaction term of treatment by sex for glucose maximum, glucose iAUC, and 60 min glucose (Table 1; *p* > 0.05). There was a noticeable difference between males and females in glycemic response to blended fruit. After consuming blended fruit, males showed a faster return to baseline with a much lower 60 min blood glucose value compared to females; however, the difference was not statistically significant (Table 1; Figure 3; mean ± SEM: 0.3 ± 3.5 mg/dL for males vs. 11.0 ± 4.2 mg/dL for females; *p* = 0.15).

## 4. Discussion

This study showed that consuming apples and blackberries that have been processed in a blender yields a reduced postprandial glycemic response compared to consuming them in whole form, as measured by glucose maximum, glucose iAUC, and 60 min glucose. The trend that we observed may be associated with the fact that we used a seeded fruit (blackberries with seeds included) that was added to a non-seeded fruit (apples with seeds removed), given the results from two other studies. Redfern et al. reported that blended mango did not have a significantly different glycemic index compared to whole mango in healthy subjects, but blended “mixed” fruit that contained mango, banana, passion fruit, pineapple, kiwi, and raspberries had a significantly lower glycemic index compared to whole mixed fruit [19]. In a follow-up study by Alkutbe et al., involving obese and non-obese subjects, they showed that consuming blended mango plus a seeded fruit (passion fruit or raspberries) significantly lowered the glycemic index (GI) compared to consuming whole mango plus a whole seeded fruit [20]. Alkutbe et al. postulate that grinding the seeds in the passion fruit and raspberries, during the blending process, may have released fiber, polyphenols, fats and proteins, which may reduce the rate of gastric emptying and glucose absorption in the small intestine [20].

We used blackberries in our fruit treatments, which are in the same genus as raspberries (*Rubus*), and they have a similar morphology to raspberries [28]. Blackberries are also high in polyphenols [21,22], which are antioxidants with many health benefits [27,28], and dietary fiber [27]. Like the Alkutbe et al. study, grinding the seeds in blackberries during the blending process may have released additional fiber, polyphenols, fats, and protein that would not otherwise be released during mastication or normal digestive processes when the fruit is consumed in whole form. Blackberry seeds contain the highest amount of polyphenols (88%) compared to other parts of the fruit (12%) [29]. Polyphenols from fruit have been shown to inhibit glucose transport (via inhibition of SGLT1 and GLUT2 transporters) from the intestine into/out of intestinal Caco-2 cells [26]. This is a potential mechanism for how polyphenols from blackberry seeds might reduce glycemic response in the “blended fruit” treatment. Further, polyphenols from berries have been shown to inhibit both α-amylase and α-glucosidase, located in the brush border of the small intestine [30], which may inhibit carbohydrate digestion and glucose availability [31], reducing the glycemic response.

The addition of fiber and protein, from the blackberry seeds to chyme, may increase chyme viscosity, decrease gastric emptying rate, and decrease the rate and degree of glucose absorption. Meal enrichment of soluble dietary fiber has been shown to increase chyme viscosity [14,15] and decrease postprandial blood glucose, fasting blood glucose, and insulin concentration [14]. Seeds from raspberries, a congener to blackberries, were 10.5% protein and 63.9% dietary fiber, with 62.7% being insoluble fiber and 1.2% being soluble fiber [32]. Even though soluble fiber is what increases chyme viscosity, not insoluble fiber, grinding insoluble fiber changes its physical properties to make it more soluble. Decreasing the particle size of coconut fiber has been shown to significantly increase its solubility [33]. Further, Liu et al. reported that grinding insoluble fiber, via ball-milling, redistributed dietary fiber components from the insoluble fiber fraction to the soluble fiber fraction, and significantly increased the ability of insoluble fiber to retard glucose diffusion and inhibit α-amylase activity by 35% [34]. In terms of the protein in blackberry seeds, adding protein to a carbohydrate meal has been shown to significantly reduce the postprandial glycemic response in healthy individuals [35,36], which might be caused by increased food viscosity from the additional protein [37]. However, it is less likely that added protein, rather than fiber, from grinding blackberry seeds, would have significantly altered glycemic response, given the low (10.5%) protein content of *Rubus* seeds [32]. Further analysis would need to be done to confirm that, in comparison to whole seeded fruit that is masticated, blended seeded fruit has (1) significantly higher soluble fiber content and (2) significantly higher viscosity.

There is one other study, that we know of, that looked at glycemic response to whole apples, blended apples, and apple juice (apple seeds were not present in the meal treatments) and it was published in 1977 [18]. Unlike the more recent studies, including our own, they measured postprandial serum insulin, in addition to plasma glucose. Haber et al. found that maximum plasma glucose and glucose area under the curve were not significantly different after consuming whole apples, blended apples, and apple juice [18]. However, they found that between 60 min and 180 min, plasma glucose values dropped significantly lower (below fasting levels) for apple juice compared to whole apple; with blended apple showing glycemic values that were intermediate between whole apples and apple juice [18]. This pattern can be explained by a higher serum insulin peak observed with apple juice compared to blended apple and whole apple; with blended apple showing a serum insulin peak that was intermediate between apple juice and whole apple [18]. Synthesizing the results from the three studies discussed [18,19,20], and our own results, we propose that consuming blended fruit without seeds (mango or apple) may not affect the glucose peak or glucose area under the curve (glycemic index) in comparison to consuming those fruits in whole form; but blending these fruits (mango or apple) may result in a higher insulin response, which could result in sub-baseline glucose values one to two hours after the meal [18]. Additional analysis of individual fruits, that include insulin response, would help to confirm this synthesis. Conversely, consuming blended fruits that contain seeds (raspberries or blackberries) will likely decrease the glucose peak, the glucose area under the curve (glycemic index), and the 60 min glucose value in comparison to consuming those fruits in whole form. When consuming mixed fruit (seeded fruits combined with non-seeded fruits), it appears that blending reduces the glycemic response similarly to blending seeded fruits (alone).

While we observed that males showed a faster return to baseline than females after consuming blended fruit, these differences were not statistically significant (*p* = 0.15). A lack of statistical significance may be due to small sample size, as we only had eight males in our study, or there may not be a statistically significant difference between males and females. Several studies have reported sex-specific differences in postprandial glycemic response to various meal treatments. Niclis et al. reported that women exhibited a lower postprandial glycemic response than men after consuming all variations of biscuits that varied in nutrient content [23]. Ahmed et al. reported a lower postprandial glucose peak in women compared to men, especially after breakfast [22]. They also reported sex-specific differences in postprandial glycemic response and insulin response associated with time of day [22]. Another study reported a lower glycemic response with increased dietary fiber intake in women but not men, and a flattening of glucose AUC with increased fat intake in women but not men [21]. Even though we did not observe significant sex-specific difference in glycemic response, sex should be considered as an independent variable in future studies that look at how different foods and food processing affect glycemic response and insulin response.

There are a few improvements that could be made in future experimental trials. We were unable to measure insulin, which is a far more invasive procedure than measuring glucose. However, it would have been useful to measure insulin response for several reasons. One reason is that avoiding chronic insulin spikes is important in preventing insulin resistance. Another reason is that it is not well understood why fruits with a similar GI can yield different insulin responses. The AUC_insulin_/AUC_glucose_ ratio has been shown to vary significantly among different fruits [38]. Whole oranges have been shown to yield a lower insulin response than orange juice while, conversely, grape juice yielded a lower insulin response than whole grapes, despite having a similar glycemic response [39]. Given that a larger volume of blood is required for insulin measurement than glucose measurement, one could take a single blood draw for insulin when it peaks at 45–60 min after a meal [22], which would, at least, provide the insulin maximum. Another improvement would be to take postprandial glucose measurements for a longer duration; at least 120 min. For most of our experimental trials, excluding blended fruit in males, postprandial blood glucose values were still falling at 60 min, and had not yet reached baseline. Glucose monitoring for 120 min would provide a more complete glycemic profile and allow one to determine if sub-baseline glycemic values at 120 min were associated with a higher insulin peak [18]. It would be valuable to separate different fruits among the whole vs. blended treatments to confirm that blending seeded fruits yields a lower glycemic response in comparison to whole seeded fruits, while blending non-seeded fruits has no effect on glycemic response. Finally, for future glycemic response trials, it would be preferrable to begin at 5:00 PM, rather than 4:00 PM, as 4–4.5 h of fasting is preferrable to 3–3.5 h of fasting. The size and nutritional profile of the participant’s lunch can vary, and this could influence the effect of treatment on glycemic response if the fasting period is too short, potentially introducing noise into the data set. However, given that we still observed a significant reduction in glycemic response in blended fruit compared to whole fruit, despite having potentially added noise, we can confidently state that a treatment effect exists.

Ultimately, our results should not be extrapolated to commercial fruit smoothies, which typically use apple juice, sorbet, or ice cream as the base, rather than water. We used water in our smoothie to control the amount of glucose present in blended fruit vs. whole fruit treatments. Adding juice or ice cream to a fruit smoothie would significantly increases the sugar content of the smoothie, without increasing fiber content, which would increase glycemic response. Haber et al. reported that apple juice yielded a significantly larger insulin response than either blended apples or whole apples [18], and thus, adding apple juice to smoothies may not be a healthy choice. Further, the serving size of commercial fruit smoothies is much larger than that of our fruit smoothies. Our fruit smoothie was only 248 mL (1 cup), to control for glucose content in whole vs. blended fruit, whereas bottled fruit smoothies sold at grocery stores and convenient stores are typically 450 mL; 81% larger than our serving size. Freshly blended fruit and vegetable smoothies sold from the popular American smoothie company, “Jamba Juice”, are 473 mL (small), 650 mL (medium) and 828 mL (large). For these reasons, one cannot infer that consuming commercial smoothies will yield a lower glycemic response than consuming whole fruit.

## 5. Conclusions

This study demonstrates that fruit smoothies, without added sugars, can be a healthy way to consume the recommended daily dose of fruits if the fruit serving size is equivalent to what one would consume if the fruit(s) were whole. Fruit smoothies containing berries, such as blackberries or raspberries, may yield a lower glycemic response than consuming those fruits whole. We cannot conclude that there are sex-specific differences in glycemic response to whole fruits vs. blended fruits, but more studies like this, with a larger sample size, are needed for a more definitive conclusion. Future studies of this nature should include measurements of (1) dietary soluble fiber, before and after blending, as blending may increase the soluble fiber fraction, and (2) peak serum insulin concentration, with the goal of understanding which foods promote hyperglycemia and hyperinsulinemia, to prevent the development of insulin resistance and type 2 diabetes in healthy individuals.

## Figures and Tables

**Table 1:** Mean glycemic response values ± SEM, with baseline glycemic values subtracted from incremental glycemic values, from 20 participants after consuming either whole fruit or blended fruit.

	Males (*n* = 8)		Females (*n* = 12)		Sex Effect	All (*n* = 20)		Treatment Effect	Treat * Sex Effect
	Whole	Blended	Whole	Blended	*p* Value	Whole	Blended	*p* Value	*p* Value
Max	48.9 ± 10.8	28.2 ± 3.6	38.2 ± 2.5	29.1 ± 3.3	0.64	42.5 ± 4.6	28.8 ± 2.4	**0.004**	0.42
iAUC	1314 ± 269	721 ± 142	1239 ± 116	935 ± 155	0.47	1269 ± 124	850 ± 109	**0.005**	0.73
60-min	11.3 ± 3.5	0.3 ± 3.5	12.6 ± 2.6	11.0 ± 4.2	0.17	12.1 ± 2.0	6.7 ± 3.0	0.057 *	0.15

“Max” is maximum blood glucose (mg/dL), “iAUC” is incremental area under the curve for glucose (mg/dL × min), and “60-min” is the incremental blood glucose value at 60 min (mg/dL). The “*p* value” columns provide *p* values from a repeated measures two-way ANOVA with treatment as the within-subjects variable and sex as the between-subjects factor. Statistically significant *p* values are bolded and marginally statistically significant *p* values have an asterisk.

## Data Availability

Restrictions apply to the availability of the raw data generated and analyzed during this study to preserve subject confidentiality. The corresponding author will, on request, explain conditions under which access to some data may be provided.

## Supplementary Materials

The following supporting information can be downloaded at https://www.mdpi.com/article/10.3390/nu14214565/s1, Protocol S1: Glucose Oxidase/Peroxidase Assay [40,41]. Figure S1: Mean incremental blood glucose values from 20 participants over 60 min, after consuming either whole fruit (solid line) or blended fruit (broken line).
