# Supplementary material for: Postprandial Glycemic Response to Whole Fruit versus Blended Fruit in Healthy, Young Adults
Source: Nutrients. 2022 Oct 30;14(21):4565. doi: 10.3390/nu14214565 (PMC9657402; doi:10.3390/nu14214565)
Supplement: Supplementary file 1 [file nutrients-14-04565-s001.zip › Figure S1.pdf]

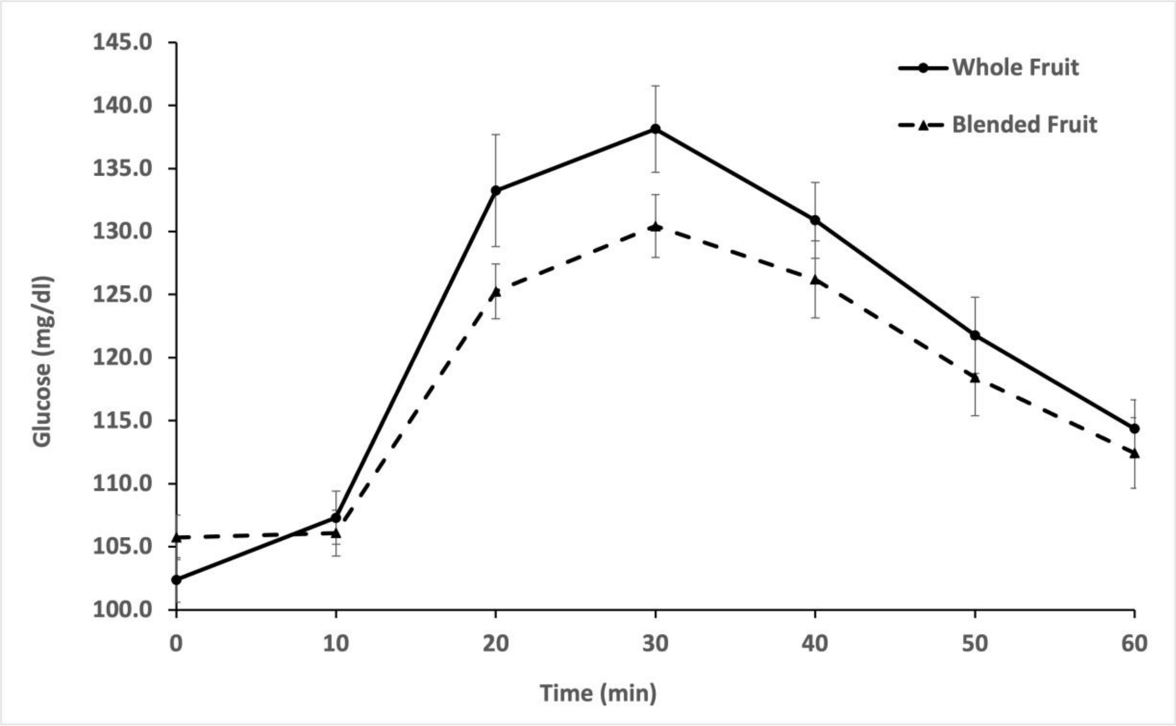

Figure S1. Mean incremental blood glucose values from 20 participants over 60 min, after consuming either whole fruit (solid line) or blended fruit (broken line). Error bars represent standard error of the mean (SEM).
