# Supplementary material for: Postprandial Glycemic Response to Whole Fruit versus Blended Fruit in Healthy, Young Adults
Source: Nutrients. 2022 Oct 30;14(21):4565. doi: 10.3390/nu14214565 (PMC9657402; doi:10.3390/nu14214565)
Supplement: Supplementary file 1 [file nutrients-14-04565-s001.zip › Protocol S1.pdf]

Glucose Oxidase/Peroxidase Assay
Modified from Dahlqvist 1968 and German and Bittong 2009.

Reagents:
Glucose Oxidase Type VII (Sigma G2133)
Peroxidase Type VI-A (Sigma P6782)
O-dianisidine dihydrochloride (Sigma D3252)
Invertase (Sigma I4504)
12N Sulfuric Acid
1M Tris-HCl, pH 7.0

Making the Glucose Oxidase/Peroxidase Reagent, o-Dianisidine and Assay Reagent
Dissolve 5.76 mg of glucose oxidase (12 U/ml) and 0.45 mg of peroxidase (5U/ml) in 100ml of 1M Tris-HCL, pH 7.0. Then prepare the o-dianisidine by dissolving 10.1 mg of o-dianisidine in 2ml of ultrapure water. Add 2ml of o-dianisidine to 100 ml of the solution containing glucose oxidase and peroxidase in Tris-HCL, pH 7.0. Store at 4°C under minimal light until use.

Assay Procedure
1. Label 1.5ml microcentrifuge tubes containing blended fruit and label tubes for each dextrose dilution for the dextrose standard curve (0mM, 2mM, 4mM, 6mM, 8mM, 10mM).
2. Pipette 10ul of each substrate and 20ul of invertase into corresponding microcentrifuge tubes. Briefly vortex tubes and incubate for 60min at 40°C.
3. Add 300ul of assay reagent to each tube, briefly vortex, and incubate for 30min at 45°C.
4. Add 300ul of 12N sulfuric acid to each tube and vortex.
5. Add 100ul of each sample in triplicate into a polypropylene microplate. In one column, pipette 100ul of each dextrose dilution in ascending order from top to bottom. Read absorbance values 540nm in a plate with the lid on the microplate.
6. Subtract the 0mM dextrose (blank) absorbance reading from the other dextrose dilution readings and the fruit sample readings.
7. Generate a standard curve of absorbance at 540nm vs. dextrose dilution and use the equation of the line to determine glucose concentration from unknown samples.

Total in each tube	Dilution of Substrate 1:63
Substrate	10ul
Invertase	20ul
Reagent	300ul
H2SO4	300ul
Total	630ul

References:
Dahlqvist, A. (1968). "Assay of intestinal disaccharidases." Analytical biochemistry **22**(1): 99-107

German, D. P. and R. A. Bittong (2009). "Digestive enzyme activities and gastrointestinal fermentation in wood-eating catfishes." Journal of Comparative Physiology B **179**(8): 1025-1042.
